# Vacuolar protein sorting mechanisms in apicomplexan parasites

**DOI:** 10.1016/j.molbiopara.2016.01.007

**Published:** 2016

**Authors:** Elena Jimenez-Ruiz, Juliette Morlon-Guyot, Wassim Daher, Markus Meissner

**Affiliations:** a Wellcome Trust Centre for Molecular Parasitology, Institute of Infection, Immunity & Inflammation, College of Medical, Veterinary and Life Sciences, Glasgow, Lanarkshire, United Kingdom; b Dynamique des Interactions Membranaires Normales et Pathologiques, UMR5235 CNRS, Université Montpellier, Montpellier, France

**Keywords:** PM, plasma membrane, IMC, inner membrane complex, PV, parasitophorous vacuole, ER, endoplasmic reticulum, TGN, trans-Golgi network, ECL, endosomal-like compartment, VAC, vacuolar compartment, PLV, plant-like vacuole, EE, early endosome, LE, late endosome, MVB, multi-vesicular body, VPS, vacuolar protein sorting, MTC, multisubunit tethering complex, GARP, Golgi-associated retrograde protein, ESCRT, endosomal sorting complex required for transport, CORVET, class C core vacuole/endosome tethering, HOPS, homotypic fusion and vacuole protein sorting, Vesicular protein sorting, *Toxoplasma gondii*, Multi-subunit tethering complex, Microneme recycling

## Abstract

The phylum Apicomplexa comprises more than 5000 species including pathogens of clinical and economical importance. These obligate intracellular parasites possess a highly complex endomembrane system to build amongst others three morphologically distinct secretory organelles: rhoptries, micronemes and dense granules. Proteins released by these organelles are essential for invasion and hijacking of the host cell. Due to the complexity of the internal organization of these parasites, a wide panoply of trafficking factors was expected to be required for the correct sorting of proteins towards the various organelles. However, *Toxoplasma gondii* and other apicomplexan parasites contain only a core set of these factors and several of the vacuolar protein sorting (VPS) homologues found in most eukaryotes have been lost in this phylum.

In this review, we will summarise our current knowledge about the role of trafficking complexes in *T. gondii*, highlighting recent studies focused on complexes formed by VPS proteins. We also present a novel, hypothetical model, suggesting the recycling of parasite membrane and micronemal proteins.

## 1 Introduction

The endomembrane system consists of various membranes in the cytoplasm, which organizes the cell into functional compartments, the organelles [1]. Each organelle possesses a specific membrane structure and function and is connected to other organelles either by direct contact or by exchanging material by vesicular transport. The organelles of the endomembrane system include the plasma membrane (PM), nuclear membrane, endoplasmic reticulum (ER), Golgi apparatus and the lysosomes, vacuoles, vesicles and endosomes. The development of a complex endomembrane system was crucial for the functional compartmentalization within eukaryotic cells and it appears that early eukaryotes possessed systems with near modern complexity prior to the radiation into major eukaryotic lineages [2]. In fact, most of the trafficking factors (i.e. Rab-GTPases, SNARES and tethers) were present in the last common eukaryotic ancestor and subsequent gene duplications or secondary loss led to complex and specialised endomembrane systems in diverse eukaryotes [3], [4].

The ER is the headquarters for cellular biosynthesis and transport and can be branched out throughout the cytoplasm. The Golgi apparatus is a set of multiple compartments where molecules are packaged with the aim of their delivery in other cellular components or are designated to be secreted [5]. The endolysosomal system controls the traffic of proteins and lipids from the plasma membrane to the lysosome (or vacuole). While the endosomes have been initially defined as compartments derived from endocytosis of material from the PM, the endosomal pathway is much more complicated than initially described and is interconnected with the secretory traffic network [6]. During their formation, endosomes undergo multiple steps, beginning from early endosomes (EE), followed by multi-vesicular bodies (MVB), also known as late endosomes (LE), until fusing with the lysosome (or vacuole). However, this overall organization of the endomembrane system underwent phylum specific modification, often reflected by the loss or expansion of trafficking factors between different phyla [4], [7], [8], leading to unique adaptations. This includes the loss of organelles or the development of unique organelles that are only present in certain phyla.

While much of our knowledge of the organization and function of the endomembrane system comes from studies in plant and ophistokonts, relatively little is known about the organization of this complex system in other eukaryotes, such as apicomplexan parasites. The Apicomplexa phylum comprises more than 5000 species including pathogens of clinical and economical importance, such as *Plasmodium* spp., *T. gondii, Eimeria* spp. and *Cryptosporidium* spp. These obligate intracellular parasites possess a highly complex endomembrane system involved, amongst others processes, in the biogenesis of the unique apicomplexan secretory organelles (micronemes, rhoptries and dense granules). In order to successfully invade the host cell, these parasites employ an arsenal of virulence factors contained in these organelles which are sequentially secreted during the invasion process and contribute to the establishment of the parasitophorous vacuole (PV) [9], [10], [11].

While the content of the secretory organelles and individual virulence factors have been well described previously [12], [13], our knowledge on the evolution, biogenesis, maintenance and regulation of these unique organelles is still immature. Intriguingly, the complex apicomplexan cellular architecture is not reflected by an expansion of trafficking factors. Instead multiple gene losses had occurred during their evolution, with some well conserved trafficking complexes being absent [8].

In this review, we will summarise our current knowledge about the role of trafficking complexes in *T. gondii*, an attractive model apicomplexan parasites and present a hypothesis for how a complex cellular architecture can evolve, concurrently with the loss of trafficking complexes.

## 2 Organization of the endomembrane system in apicomplexans

The apicomplexan secretory system is highly polarised, consisting of the ER, a single Golgi stack [14] and (ill-defined) endosomal-like compartment (ELC) and a vacuole-like compartment termed plant-like vacuole (PLV) or vacuolar compartment (VAC) [15], [16], [17], [18].

Unique secretory organelles, micronemes and rhoptries can be identified at the apical pole of the parasite, whereas dense granules can be found more evenly distributed within the parasite [19]. The elaborate alveolar system, called the inner membrane complex (IMC) [20] and the apicoplast (a chloroplast-like organelle) [21], [22], are also directly linked to the secretory system.

Other organelles have been described in apicomplexans and are probably connected to the secretory system including the acidocalcisomes (calcium storage organelles [23]), exonemes (secretory organelles involved in host cell egress in *Plasmodium* [24]) and osmiophilic bodies (secretory organelles identified in *Plasmodium* gametocytes [25]).

Secretory proteins co-translationally enter the ER via the signal recognition particle (SRP) pathway [26]. Subsequently proteins are trafficked specifically to their final destination and some sorting signals have been identified for trafficking to the micronemes and rhoptries. Rhoptry proteins are transported from the Golgi to endosomal like compartments and premature rhoptry compartments where processing can occur [27]. For example, the rhoptry protein ROP2 has been previously characterised as a transmembrane protein and a di-leucine motif in the cytosolic tail has been implicated in transport to the rhoptries via interaction with the adaptor complex 1 (AP1) [28], [29]. Intriguingly, recent data demonstrated that ROP2 is a soluble protein, meaning that no direct interaction between AP1 and ROP2 is possible [30]. Therefore the mechanism involved in protein transport to the rhoptries requires further, detailed analysis.

Similar to rhoptry proteins, pro-peptide processing appears to be a common feature for micronemal proteins during their transport through the secretory pathway and this processing appears to occur *en-route* in the VAC/PLV [16], [17]. Some micronemal transmembrane proteins contain tyrosine-based signals in their cytosolic tails, as shown for MIC2 and MIC6 that are required for their transport to the micronemes [31], [32]. Soluble microneme proteins appear to be transported in complexes with transmembrane proteins, as shown for MIC6-1-4 and MIC2-M2AP [17], [31].

Interestingly, not all micronemal transmembrane proteins, such as MIC8 or AMA1 [33], [34] contain a well-defined sorting signal in their cytosolic tail for their transport to the micronemes. It has been demonstrated that these proteins are transported in a complex with other proteins to their final destination (mature microneme organelles) [34], our recent analysis indicates that these same proteins are targeted at the end of their journey to different subsets of micronemes [35]. These subsets do not only have different protein content, they also employ an independent transport pathway, since transport of MIC8, MIC3 and MIC11 depends on the Rab-GTPases Rab5A and C, while transport of MIC2, MIC6 and AMA1 can occur independently of these small GTPases. Intriguingly, rhoptry protein transport appears to depend on Rab5A and C as well, suggesting that the specific transport to micronemes and rhoptries is interconnected with the endosomal-system [35].

## 3 The enigmatic endosomal system of *T*. *gondii*

To date it is still unclear if and to what extent *T. gondii* has an operational endocytic system. While two reports suggest that the parasite shows a certain activity of receptor mediated endocytosis [36], [37], our functional characterisation of factors typically involved in endocytosis, such as clathrin, dynamin or Rab-GTPases failed to convincingly demonstrate an involvement in this process thus far [35], [38], [39], [40], [41], [42]. Instead, conditional mutants for these trafficking components demonstrated impressive phenotypes regarding protein transport to the unique secretory organelles or the IMC, leading to the hypothesis that apicomplexans re-shaped their endocytic system in order to develop these unique organelles [15], [35]. However, in a series of elegant experiments, the Carruthers group recently demonstrated not only uptake of host cell proteins by the parasite, but also suggested the involvement of the endocytic system in their digestion [18]. When parasites deficient in the cathepsin protease L (CPL) were cultivated on host cells expressing cytosolic GFP, a strong accumulation of GFP was observed in the VAC/PLV. Furthermore this organelle associates with markers of the late endocytic pathways, such as Rab7 [16], [23]. Although the exact mechanism of uptake remains to be characterised, these exciting findings will lead to a detailed understanding of the potentially dual functions of trafficking components in uptake and/or secretion of proteins.

Endosomes are sorting organelles at the intersection between secretory and endocytic traffic in diverse eukaryotic phyla [43]. They receive and send vesicles from the trans-Golgi network (TGN), the plasma membrane and the lysosome. While in ophistokonts, the general pathway can be described as a stepwise transport from EE to LE and then to the lysosome [44], EE have so far not been discovered in plants [45]. Instead an elaborate TGN appears to directly receive endocytic vesicles from the PM and the Golgi. Interestingly, Syntaxin 6 (Stx6), a marker of the early endocytic system, localises to distal Golgi cisternae, indicating that the apicomplexan endosomal system might have a plant-like configuration, with a more elaborated TGN than previously thought [39].

## 4 Endosomal factors involved in the biogenesis of secretory organelles

**Fig. 1:**
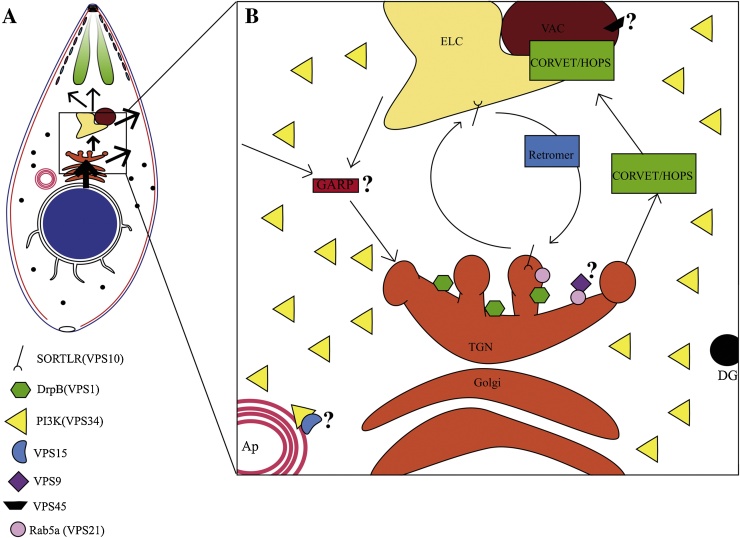
Vesicular trafficking in *Toxoplasma gondii*. (A) Classical unidirectional model for transport of proteins from ER to secretory organelles via endosome like compartment (ELC) and vacuolar compartment (VAC). (B) Detail of trafficking between Golgi and endosomal compartments and the position of multisubunit tethering complexes formed by VPS proteins.?: unknown interaction, localisation and/or function; Ap: apicoplast; DG: dense granules; TGN: trans Golgi network; SORTLR: sortilin-like receptor; DrpB: Dynamin-like protein B; PI3K: phosphatidylinositol 3-kinase.

Much of our knowledge on the general organisation of endosomal transport complexes in eukaryotes has been derived from forward genetic screens performed in yeast more than 20 years ago aimed at identifying transport defects of carboxypeptidase Y (CPY) to the yeast vacuole, which is analogous to the lysosome. To date more than 70 different vacuolar protein sorting (VPS) mutants have been identified [46] and grouped into 6 classes (A–F) [47]. Subsequent analysis demonstrated that proteins of one class are functionally linked and often form a complex. Most of the VPS proteins are organised in 4 major multi-subunit complexes which play a critical role in vesicular transport to, within and from the endosomes in yeast and other eukaryotes (Table 1; Fig. 1):

1. Retromer complex is required for retrograde transport from the endosomes to Golgi and for a recycling route from endosomes to the cell surface in higher eukaryotes [48].
2. HOPS/CORVET are required for transport within the endosomes (EE to LE to lysosomes; TGN to lysosomes) [49].
3. GARP is required for transport of vesicles from the endosomes to the TGN [5].
4. ESCRT-complexes are required for organisation of multivesicular bodies (MVB), endocytosis and cytokinesis [50].

Interestingly, a recent analysis suggests that the proto-apicomplexan had a nearly complete repertoire of the endomembrane trafficking complexes, while differentiation of apicomplexan lineages was accompanied by lineage-specific losses (Table 1; [8]). However, while comparative genomics allows us to speculate on modifications and alterations of the apicomplexan endomembrane system, a detailed functional analysis of key-trafficking factors is required. This will allow to test the hypothesis that the reduced repertoire of trafficking factors in apicomplexans has been compensated by evolutionary re-purposing of the endosomal system to enable the evolution of an elaborate secretory system [51]. Here we summarise the current efforts to understand the role of the different tethering factors mentioned above and present a novel, hypothetical model, suggesting the continuous recycling of some microneme proteins.

### 4.1 ESCRT machinery in apicomplexan parasites

The endosomal sorting complexes required for transport (ESCRT) machinery is a set of 5 sub-complexes involved in MVB biogenesis through the formation of intraluminal vesicles. These complexes recognise ubiquitinylated proteins and recruit them to endosomes for degradation. Another important function of these complexes is ESCRT-mediated membrane scission facilitating changes in membrane architecture [52]. However, very little is known about the role of ESCRT subunits in apicomplexan parasites.

While most eukaryotes have a complete set of the ESCRT machinery, apicomplexan parasites exhibit significant reductions in their ESCRT machinery [8]. In fact, ESCRTIII is the only complex that appears to be present in all apicomplexans, while ESCRTI and II are lost in several apicomplexans, such as *Plasmodium spp.* or *T. gondii* (Table 1). Interestingly, ESCRT-III is involved in functions unrelated to vesicular transport or endocytosis, such as sealing and reorganisation of the nuclear envelope during cytokinesis [53], [54]. It is thus possible that ESCRTIII was retained to ensure essential functions unrelated to endocytosis or vesicular transport. One of the ESCRTIII components, VPS4, is an AAA ATPase that contributes directly to the ESCRTIII-mediated membrane remodelling by completing fission of membranes [50]. In *Plasmodium falciparum*, VPS4 seems to localise in the cytoplasm during the trophozoite stage [55]. Transfection of an inactive form of the protein (VPS4^E214Q^) in *T. gondii* leads to an alteration of the endolysosomal system indicated by the formation of MVB-like structures, suggesting a conserved function of Vps4 in organisation of the endosomal system.

### 4.2 VPS tethering complexes in apicomplexan parasites

Multi-subunit tethering complexes (MTC) are heterodimeric complexes involved in the docking of transported vesicles to their acceptor compartment [56]. In general, MTCs seem to couple the recognition of membranes via Rab GTPases with subsequent SNARE-mediated membrane fusion. Amongst the MTCs, VPS homologues can be identified in *T. gondii* for: GARP, Retromer and CORVET/HOPS complexes.

### 4.2.1 GARP

The heterotetrameric tethering factor named Golgi-associated retrograde protein (GARP) promotes fusion of endosome-derived and retrograde transport carriers to the TGN. The GARP tethering complex consist of 4 subunits (VPS51, VPS52, VPS53 and VPS54) which seem to cooperate with the RabGTPase Ypt6/Rab6 (via VPS52) in tethering endosome-derived vesicles to the TGN [57] (Fig. 1B). Vps51 specifically binds to the yeast SNARE Tlg1 [58] and to Stx6 in mammalian cells [59]. While no functional data exist for VPS51-54 in *Toxoplasma,* Stx6 appears to have a role in retrograde transport from the ELC to the Golgi and interference with Stx6 function does not directly affect the transport of proteins to secretory organelles but seems to impair IMC maturation in budding daughter cells [39]. Furthermore, dense granules are also affected by the overexpression of Stx6, similar to functional interference with TgRab6, a small GTPase that is localised at the TGN [60]. While these findings suggest a conserved role of GARP in *T. gondii* further functional analysis is required.

### 4.2.2 Retromer

The major role of the retromer complex is recycling cargo receptors from the endosomes to the Golgi and PM to the Golgi. PM receptors recycling has been recently described in higher eukaryotes [48]. This recycling is mediated through interaction of retromer with another macromolecular complex: WASH. This protein complex facilitates endosomal protein sorting by stimulating Arp2/3 to nucleate F-actin on endosomal membranes. Since the Arp2/3 nucleator complex is absent in apicomplexan parasites [61], the classical endosome-to-PM recycling pathway might have been lost in these organisms and an alternative form of PM recycling, possibly dependent on microtubules, has evolved in apicomplexan parasites. Nonetheless, it would be interesting to investigate the role of actin in endosomal trafficking in apicomplexan parasites.

The retromer complex is formed by the proteins VPS26, VPS29 and VPS35 that are conserved in *T. gondii* and *Plasmodium* (Table 1; [8], [62]). In higher eukaryotes, retromer recycles the cargo receptor VPS10/sortilin from endosomes to the Golgi [63], [64]. This cargo receptor is a transmembrane protein which target soluble proteins, such as CPY, pro-proteinase A and Aminopeptidase Y, to the vacuole in yeast [65]. In recent studies, homologues of VPS10 have been described in *T. gondii* and *P. falciparum* [62], [66]. The sortilin-like receptor (SORTLR) has been localised to the Golgi and found to interact with several MIC and ROP proteins for their transport to the ELC (Fig. 1B). The interaction of the retromer complex with the cytoplasmic tail of SORTLR has been confirmed in *T. gondii* [66].

Knockdowns for components of retromer and TgSORTLR in *T. gondii* have demonstrated an abnormal localisation of microneme, rhoptry and dense granule proteins. These findings highlight the essentiality of cargo receptors recycling for trafficking towards the secretory organelles as well as for the biogenesis of micronemes, rhoptries and dense granules [66], [67], [68].

### 4.2.3 HOPS/CORVET

In yeast and mammalian cells, the fusion of membranes within the endolysosomal system requires a conserved machinery which involves two tethering complexes called CORVET (class C core vacuole/endosome tethering) and HOPS (homotypic fusion and vacuole protein sorting) [69]. CORVET is mainly associated with EE and HOPS with LE/MVB and lysosomes/vacuole. Both complexes share four subunits (the Vps-C core), and additional compartment-specific subunits (VPS3 and VPS8 in CORVET and VPS39 and VPS41 in HOPS; Table 1) [6]. CORVET is a Rab5 effector complex, whereas HOPS can bind efficiently to late endosomes and lysosomes through Rab7 [69]. VPS11 occupies a central position in the Vps-C core and its homologue in *T. gondii* has been characterized and associates with TGN, ELC, VAC and immature apical secretory organelles (Fig. 1B) [67]. VPS11 is also essential for the biogenesis of secretory organelles and required for the proper subcellular localization of micronemes, rhoptries and dense granules. Besides, VPS11 is required for the correct localisation of its interaction partners, such as Rab7 [67], suggesting the importance of these complexes in the maintenance of endolysosomal integrity.

In *T*. *gondii* VPS11-depleted parasites, several microneme, rhoptry and dense granule proteins were not correctly targeted to their respective organelles and were secreted constitutively into the PV. However, AMA1 and MIC2 proteins showed an apical localisation in those mutants indicating that at least two different populations of micronemes and/or different trafficking pathways for micronemal proteins are present in apicomplexan parasites. This conclusion, also raised by Kremer et al., agrees with the possibility of an alternative trafficking of recycled MIC proteins and PM.

Interestingly, the phenotypic consequences of interfering with DrpB (VPS1), TgSORTLR (VPS10) or VPS11 are almost identical, suggesting that independent complexes act in a concerted manner during vesicle formation, transport and fusion required for the biogenesis of secretory organelles. In good agreement, the TgSORTLR cytoplasmic tail is bound to parasite homologues of clathrin heavy chain, the retromer complex, VPS9 and different components of the AP1 and AP2 adaptor complexes [66]; Fig. 1B). On late endosomes, Rab7-GTP can bind the membrane-remodeling retromer complex and might thus support the recycling of receptors from late endosomes [70], [71]. We noticed that the labelling of TgVPS35 (a key member of the retromer complex) is affected in the absence of TgVPS11 [67] suggesting a probable crosstalk between the retromer complex and the HOPS-like complex.

Furthermore, it will be interesting to verify if the homologue of Yck3 kinase in Apicomplexa is involved in controlling the binding of apicomplexan parasites VPS41 homologue to membranes and if additional regulatory circuits beyond casein-kinase-I-mediated phosphorylation of yeast VPS41 will control the functions of specific subunits of the CORVET/HOPS complexes [69], [72].

## 5 Is there a secretory-endocytic cycle in apicomplexan parasites?

**Fig. 2:**
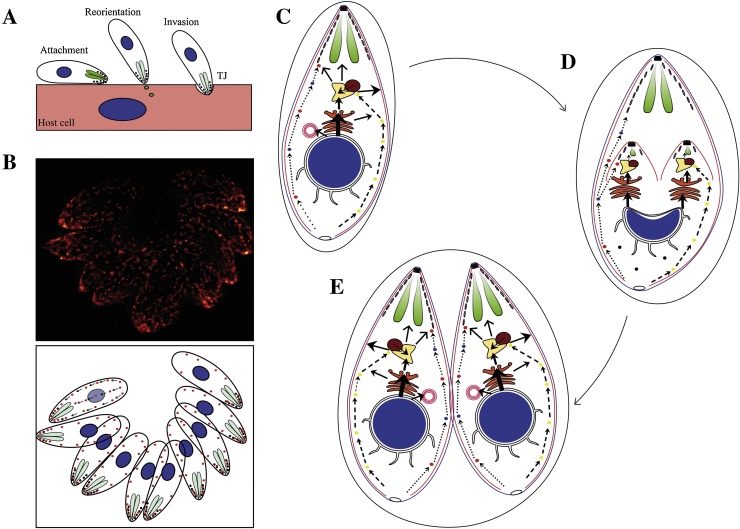
Hypothetical model of microneme protein recycling. (A) *T*. *gondii* secretes microneme, rhoptry and dense granules proteins during the process of invasion. TJ: tight junction. (B) STED image clearly suggest a highly dynamic movement of microneme organelles in *T.gondii*. Cartoon representation of the recycling process in the lower panel α-MIC2 antibodies were used for the staining of micronemes in this STED image [35]. (C–D) Parasite replication inside the host cell. (C) Micronemes might be recycled through the posterior end to apical pole (red and blue dots and dotted arrows), however only new synthesised proteins would be transported via endosomes (thick solid arrows). Host proteins and macromolecules ingested by the parasite [18] could be introduced into the parasite using this transport (yellow dots and dashed arrows). Apicoplast proteins are mainly nuclear encoded and post-translationally imported via the secretory system; however, the mechanism by which proteins are transferred from the secretory system to the apicoplast is poorly understood and probably depends on VPS34/VPS15 complex. (D) During endodyogeny microneme, rhoptry and dense granules organelles are synthesized de-novo. The recycled MIC proteins are redirected to the newly formed daughter cells along with host proteins and macromolecules ingested by the parasite (red/blue and yellow dots; dotted and dashed arrows respectively). This model would explain STED images and some conditional mutants, such as VPS11 knockdown and Rab5 overexpression mutants [35], [67] data to date. (For interpretation of the references to colour in this figure legend, the reader is referred to the web version of this article.)

With the discovery of an uptake mechanism for host cell proteins [18] it becomes attractive to speculate on the presence of a secretory-endocytic-cycle that is important for ensuring plasma membrane balance and recycling of surface proteins, such as micronemal proteins (Fig. 2B). It would also offer an attractive explanation how micronemes are formed during the intracellular development of the parasite (Fig. 2C–E). According to this model, micronemes of the daughter cells are formed *d*e novo and by recycling of micronemal proteins secreted by the mother cell via an endocytic route (Fig. 2D). Result to our hypothetical model, novel and recycled material may intersect at the EE/LE. The potential presence of a secretory/endocytic cycle might also explain the dependency of some micronemal proteins on functional Rab5A and Rab5C (Rab-GTPases typically required for endocytosis) [35].

Similarly, a secretory-endocytic-cycle might be important to ensure a residual rate of invasion and gliding motility processes in mutant parasites depleted of either rhomboid proteases or affected in microneme secretion. During parasite motility and host cell invasion multiple signalling cascades trigger the secretion of micronemes [73], resulting in deployment of the micronemal transmembrane proteins, such as MIC2 to the surface of the parasite, where they are critical to form attachment sites and thought to transmit the force generated by the parasites actin–myosin-system [74] (Fig. 2A). This polarized secretion of micronemes will not only result in the deployment of micronemal proteins, but also in the expansion of the plasma membrane at the apical pole. In order to maintain its shape the parasite most likely needs to balance this extra membrane material, either by membrane shedding or recycling. This situation is very similar to other motility systems and several reports demonstrate important roles of membrane transport and membrane flow in cell migration and the importance of a polarized exocytic-endocytic cycle during motility [75], [76], [77]. Indeed it has been speculated that motility can be driven purely by lipid flow in some cells. In this case, a secretory-endocytic cycle would act as a fluid drive and is sufficient for movement on its own [77]. In support of such an endocytic cycle, all motile cells show a distinct capping activity of surface ligands [78]. It is thus possible that membrane transport and retrograde flow during motility is rate-limiting for cell forward translocation [79].

However, to date, the regulated exocytosis of micronemal proteins at the apical end of the parasites and their redistribution toward the posterior pole is not associated with any known mechanism of membrane recycling by endocytosis. While it has been suggested that putative endocytic events (uptake of molecules via receptor-specific or fluid-phase endocytic mechanisms) take place at the micropore(s) or at the anterior third of the parasite and it has been demonstrated that the endocytosed material is degraded within a digestive compartment [18], [36], [37], the molecular mechanism for this uptake is currently unknown. In most eukaryotes endocytic uptake depends on actin and actin-binding proteins [80]. For example, the mouse coronin regulate endocytosis and membrane recycling processes [81]. Interestingly, *Toxoplasma* coronin relocalizes at the posterior end in parasites triggered for microneme secretion. This suggests a putative role of coronin protein in maintaining the integrity of the parasite pellicle by eliminating excess of membranes and micronemal proteins discharged at the apical pole [82]. However, more direct evidences are required to demonstrate the presence of a secretory-endocytic-cycle in apicomplexans and its importance for gliding motility and host cell invasion.

## 6 Conclusion

The combination of comparative genome analysis, reverse genetics, novel microscopy techniques (such as STED or SIM) and biochemistry allowed the establishment of the first models for the organisation of vesicular trafficking pathways in *T. gondii.* While many factors appear to be conserved, it seems that apicomplexan parasites reshaped the organisation of the endocytic and secretory system. Despite loss of several trafficking complexes, they allowed for the evolution of unique secretory organelles. Interestingly, recent studies strongly suggest the presence of endocytic and recycling pathways and it will now be important to consolidate these data in future studies. New technologies, such as CRISPR/cas9 will allow for rapid generation of conditional mutants for trafficking factors and the systematic analysis of their phenotypes. However, in order to analyse the role of individual proteins in more detail, rapid regulation might be important, such as *knock sideways* that allows the inactivation of protein function within seconds [83].

## Figures and Tables

**Table 1:** Vacuolar protein sorting (VPS) proteins described in yeast.

VPS	Class^a^	Complex^b^	Function/other name^c^	Localisation^d^	*T. gondii*^e^	*Plasmodium*^e^	Reference
**VPS10**	A	–	Cargo receptor between Golgi and ELC- biogenesis of micronemes and rhoptries/sortilin-like receptor	Golgi and ELC	√	√	[62], [66]

**VPS26**	A	Retromer	ELC to Golgi	ELC	√	√	[62], [67], [85]
**VPS29**	A				√	√	
**VPS35**	A				√	√	

**VPS51**	B	GARP	Endosomes to Golgi	–	√	√	–
**VPS52**	B				√	√	
**VPS53**	B				√	√	
**VPS54**	B				√		

**VPS11**	C	Vps-C core	Within endosomes	TGN, ELC, VAC and immature apical secretory organelles	√	√	[67]
**VPS16**	C				√	√	
**VPS18**	C				√	√	
**VPS33**	C				√	√	
**VPS3**	D	CORVET					
**VPS8**	A/D*				√	√	–
**VPS39**	B	HOPS			√	√	[67]
**VPS41**	B				√	√	–

**VPS9**	D	–	Activation of Rab5 (?)/GEF	Apical to the nucleus	√	√	[67]

**VPS45**	D	–	Vesicle docking and fusion at vacuole (?)	–	√		–

**VPS21**	D		Transport between Golgi and ELC/Rab5a	Golgi	√	√	[35], [86]

**VPS15**	D	–	Apicoplast homeostasis (?)	–	√	√	–

**VPS34**	D	–	Apicoplast homeostasis/PI3K	Cytoplasm	√	√	[87]

**VPS23**	E	ESCRTI	Transport to LE/MVB	–			–
**VPS28**	E						
**VPS37**	E						

**VPS22**	E	ESCRTII	Transport to LE/MVB	–			–
**VPS25**	E						
**VPS36**	E						

**VPS2**	E	ESCRTIII	LE/MVB formation and cytokinesis (?)	–	√	√	–
**VPS4**	E				√	√	
**VPS31**	E				√	√	
**VPS32**	E				√	√	
**VPS46**	E				√		
**VPS60**	E					√	

**VPS1**	F	–	Transport from Golgi to ELC/DrpB	Golgi	√	√	[42]

*VPS8 was classified in two different classes [84]. Most of the VPS mutants, class A, contained vacuoles that appeared similar or slightly perturbed compared with the wild-type cells. Mutants categorised as class B contained a fragmented vacuole appearing as numerous small vacuole-like compartments. Class C mutants, exhibited the most extreme defect in the morphology of the vacuole and appeared to lack vacuoles altogether. Mutants in Class D present a deficient inheritance and acidification of the vacuole. Class E mutants display a novel prominent prevacuolar-like organelle in yeast. Vacuoles in class F vps mutants are encircled by smaller vacuolar compartments [47].

a They were classified in six classes depending on the phenotype showed by yeast mutants.

b Most of these VPS proteins are subunits of multi-subunit tethering complexes indicated in this column.

c Function in apicomplexan parasites and alternative name is shown in this column. (?) indicates the possible function based on other eukaryotes since is still unknown for apicomplexan parasites.

d localisation in *T*. *gondii* or *Plasmodium* if known.

e Not all VPSs are present in apicomplexan parasites, we indicated the ones present in *T*. *gondii* and Plasmodium genome based on a recent study by Woo et al. [8].
